# Chitosan/Calcium-Coated Ginsenoside Rb1 Phosphate Flower-like Microparticles as an Adjuvant to Enhance Immune Responses

**DOI:** 10.3390/vetsci9070355

**Published:** 2022-07-13

**Authors:** Xinghui Song, Huijuan Li, Liheng Zhang, Xiaozhan Zhang, Li Zhao, Gaiping Zhang, Shengbo Cao, Yunchao Liu

**Affiliations:** 1 State Key Laboratory of Agricultural Microbiology, Huazhong Agricultural University, Wuhan 430070, China; sxh811520@163.com (X.S.); lihj2022@126.com (H.L.); 80206@hnuahe.edu.cn (L.Z.); sbcao@mail.hzau.edu.cn (S.C.); 2 College of Veterinary Medicine, Henan University of Animal Husbandry and Economy, Zhengzhou 450046, China; xiaozhan063@163.com (X.Z.); 80289@hnuahe.edu.cn (L.Z.); 3 Henan Academy of Agricultural Sciences, Zhengzhou 450046, China; zhanggaip@126.com

**Keywords:** GRb1, IL-4, CS/CaP, infectious bursal disease virus, vaccine, adjuvant

## Abstract

**Simple Summary:**

The field-level control over IBD is primarily via vaccination. The development of high effective IBV vaccine has drawing great attentions worldwide. Herein, the GRb1 was encap-sulated into Calcium phosphate and chitosan core-structure nanoparticles microspheres, which con-stitute a novel system for nanoparticle delivery (GRb1/IL-4@CS/Cap). The new nano-adjuvant de-livery system could induce the activation of chicken dendritic cells ( DCs ), with up-regulate the expression of MHC II and CD80, and increase the production of IL-1β and TNF-α. At the same time, it can trigger higher levels of IBDV-specific IgG and higher IgG2a/IgG1 ratio, and promote the production of IFN-γ, TNF-α, IL-4, IL-6, IL-1α, and IL-1βand other cytokines in chicken serum after vaccination, it provides an effective adjuvant system for the development of chicken IBDV attenu-ated vaccine.

**Abstract:**

Infectious bursal disease (IBD) is a highly contagious immunocompromising disorder that caused great economic losses in the poultry industry. The field-level control over IBD is primarily via vaccination. The development of a highly effective IBV vaccine has drawn great attention worldwide. Chitosan/Calcium Phosphate (CS/CaP) nanoparticle was a newly developed effective biological delivery system for drug and antigen. Ginsenoside Rb1 is one of the main bioactive components of ginseng root extract, which has antioxidant, anti-inflammatory and immunological enhancement effects. Until now, the combined effect of CS/CaP and ginsenoside Rb1 on the chicken immune response had remained unknown. In this study, the GRb1 and IL-4 were encapsulated into Calcium phosphate and chitosan core structure nanoparticles microspheres (GRb1/IL-4@CS/CaP), and the effect of a newly developed delivery system on an infectious bursal disease virus (IBDV) attenuated vaccine was further evaluated. The results demonstrated that GRb1/IL-4@CS/CaP treatment could induce the activation of chicken dendritic cells (DCs), with the upregulated expression of MHCII and CD80, and the increased production of IL-1β and TNF-α. Importantly, GRb1/IL-4@CS/CaP could trigger a higher level of IBDV-specific IgG and a higher ratio of IgG2a/IgG1 than the traditional adjuvant groups, promoting the production of cytokine, including IFN-γ, TNF-α, IL-4, IL-6, IL-1α, and IL-1β, in chicken serum after 28 d and 42 d post-vaccine. Taken in all, GRb1/IL-4@CS/CaP could elicit prolonged vigorous immune responses for IBDV attenuated vaccine in chicken, which might provide an effective adjuvant system for avian vaccine development.

## 1. Introduction

Infectious bursal disease (IBD) is a highly contagious immunocompromising disorder caused by the IBD virus (IBDV) that affects young chickens on a global scale [1]. The etiological agent IBDV belongs to the *Birnaviridae* family and disrupts the premature precursors of B-lymphocytes in the bursa of Fabricius [2]. Under environmental extremities, IBDV is stable and withstands a few disinfectant therapies. Presently, field-level control over IBD is achieved primarily via conventional vaccination [3]. Although the inactivated vaccines are potent against IBDV, the IBDV-positive chickens become severely immunocompromised, which makes them more susceptible to other conditions and less responsive to vaccines [4]. Meanwhile, apart from strengthening the chickens’ immunogenicity and protective capacity, several inactivated IBDV vaccine-containing adjuvants also enhance the chickens’ protective competence against drift IBDV variants. Therefore, an increasing amount of research is directed towards designing more efficacious and safer adjuvants, with a view to eliciting potent humoral immune responses and prompting cellular immune responses.

Based on the process of clinical medicine, the technological evolutions in vaccination have promoted safer recombinant proteins and their subunits in substitution for conventional vaccines [5]. However, protein-based vaccines yield poor clinical outcomes due to weak stability and immunogenicity, which necessitates the incorporation of adequate adjuvants for achieving vaccine efficacy enhancement [6]. Traditional vaccine adjuvants, such as aluminum adjuvants, and combined vaccines have been widely used to prevent the virus in veterinary clinical settings [7]. However, traditional vaccine adjuvants have some disadvantages, including adverse events, ineffective cellular immunity, as well as safety risks [8]. Above all, a new vaccine adjuvant is important to work for virus control at present.

Chitosan (CS) is a natural cationic polysaccharide, owing to its desirable biocompatibility, biodegradability, and powerful absorbability for innumerable pharmaceutical molecules, the application potential of chitosan (CS) in drug delivery systems is outstanding [9]. However, CS readily forms diverse shapes during the preparation process, such as nanoparticles [10], hydrogels [11], and microspheres [12]. Compared to other inorganic materials, the physical traits of CS, a natural biological macromolecule, are reasonably worse, including weak thermostability, low surface area, and poor mechanical performance, which compromise the drug delivery effect of pure CS. With such advantages as low cost of production, inherent biocompatibility, and absorption, calcium phosphate (CaP) has been extensively applied in drug delivery, which is an inorganic substance widely present in nature [13]. The CaP nanoparticle-based delivery systems were capable of preventing the deterioration of polysaccharides and antigens and initiating the humoral immune response, as suggested by a growing number of studies. Besides, although the outperformance of cationic polymers over anionic polymers has been reported by plentiful studies regarding immune response initiation and elicitation in dendritic cells (DCs) and macrophages [14], their usage in drug delivery is seriously restricted by the brittleness and rigidity of CaP. Thus, it is generally necessary to hybridize the CaP with other polymers. Hybrid CS/CaP composites, as an example, are often fabricated owing to their ability to offer many outstanding performances apart from overcoming the brittleness and rigidity of CaP.

Ginsenosides, which are the primary bioactive constituents of ginseng root extracts, have some biological effects, such as antioxidant [15], anti-inflammatory [16], and immunological enhancement effects [17]. The immune response strengthening effect of Ginsenoside Rb1(GRb1), the richest ginsenoside contained in *Panax quinquefolium* L., has been confirmed, which is a potential adjuvant that stimulates both Th (T helper)1 and Th2 responses [18,19]. The immunopotentiation function of GRb1 has been reported by prior work, which is achieved through the stimulation of diverse immunocytes, including dendritic cells and macrophages [20]. GRb1 can differentially regulate the NF-κB and PI3K/Akt/mTOR axes, thereby modulating the inherent immunity in macrophages [21]. Moreover, cellular IL-4 has been identified to process an immunomodulatory effect on macrophages cells [22].

In this study, GRb1 and IL-4 were successfully encapsulated into CS/CaP microparticles. A stable and distinctly characteristic delivery system for cationic nanoparticles (GRb1/IL-4@CS/CaP) was developed, the IBDV antigen was then covalently coupled with GRb1/IL-4@CS/CaP microparticles, and the potent function as an adjuvant was further investigated.

## 2. Materials and Methods

### 2.1. Reagents

In the current work, DMEM (Dulbecco’s Modified Eagle Medium) was procured from BioWhittaker (Walkersville, MD, USA). DMEM powder, FBS (fetal bovine serum), and trypsin-EDTA (trypsin-ethylenediamine tetraacetate) were products of Gibco BRL (Grand Island, CA, USA). Pharmaceutically pure (95%) deacetylated CS (viscosity–average molecular weight: 1.2 × 10^5^) was provided by Aoxing Biotechnology (Taizhou, China). Besides, sodium tripolyphosphate (Na5P3O10, TPP) with a purity of 85% was procured from Acros Organics, Analytical grade calcium chloride dehydrates (CaCl_2_·2H_2_O) were gained from Zhanyun Chemical (Shanghai, China); IL-4 excipients from QIAGEN (Redwood City, CA, USA). ELISA kits for cytokines (IFN-γ, TNF-α, IL-4, IL-6, IL-1α, and IL-1β) were purchased from Shanghai Langton Biotechnology Co., Ltd. (Shanghai, China). Anti-CD11c–Cy7, anti-MHC-II–APC and anti-CD80–FITC were gained from Shanghai Yubo Biotechnology Co., Ltd. Ginsenoside Rb1 was jointly developed by the Beijing NorthWeiye Metrology Institute and Beijing Century Aoko Biotechnology Co., Ltd. (Beijing, China), with a purity of 98% (CAS:41753-43-9); the chemical structural formula is shown in Figure S1. In addition, the remaining reagents and solvents used came from Sinopharm Chemical Reagent Co., Ltd. (Shanghai, China).

### 2.2. Preparation of CS/CaP Flower-like Microparticles and GRb1/IL-4@CS/CaP Microparticles

Fabrication of CS/CaP flower-like microparticles was accomplished by using a somewhat modified version of a formerly described approach [23]. Briefly, The initial step was the dissolution of CS (100 mg) in 1 wt % acetic acid buffer (20 mL) by magnetic stirring at 5 mg/mL. Then, the CS solution was added with 10 mL of TPP solution (250 mg/mL) under 20 min of stirring, and, subsequently, the resulting mixture was added with 42 mL of CaCl2 solution (180 mM) under another 10 min of stirring at ambient temperature. For the collection of microparticles, they were centrifuged at 8000 rpm for 15 min, and washed three times with 10 mL of distilled water. After placing the resulting flower-like CS/CaP microparticles (approximately 0.5 g) in 100 mL of GRb1 and IL-4 in 30% (*v*/*v*) ethanol solution, they were stirred by magnetic force for 24 h, precipitated as GRb1/IL-4 @CS/CaP particles and dried at 40 °C. HPLC was employed to determine the GRb1 residue in the solution at 203 nm. The computational formula for EE (encapsulation efficiency) of GRb1/IL-4@CS/CaP microparticles was as follows:

EE% = (M0 − M1)/M0 × 100%(1)

where M0 represents the total amount of added GRb1, and M1 stands for the amount of unencapsulated free GRb1. In this work, three replicate tests were carried out in the research on drug loading.

### 2.3. Measurement of Particle Size of GRb1/IL-4@CS/CaP Microparticles

Following the incorporation of 10% FBS and transient ice preservation (for avoiding particle aggregation), Zetasizer (Nano ZS, Malvern, UK) was adopted for examining the size of the GRb1/IL-4@CS/CaP microparticles. A JEM-6360 SEM (scanning electron microscope) was utilized to accomplish morphological observation of the CS/CaP microparticles.

### 2.4. Animal Immunization

One-day-old SPF chickens were purchased from the Ji’nan Sipsifu Poultry FARM Technology Co., Ltd. (Jinan, China). Through adaptive feeding a week before immunization, the first three days of temperature control at about 35 °C, from the fourth day, the daily body temperature decreased by 3 °C to 26 °C. The present experimental protocols were all supported by the Ethics Committee of the China Animal Health and Epidemiology Center in Zhengzhou, China. Apart from that, the IBDV strain and chicken experiments were all carried out at a China Animal Health and Epidemiology Center’s BSL-3 (biosafety level 3) facility, which is equipped with a highly efficient particulate air-filtered isolator.

We collected the IBDV antigens from the IBDV-cocultured embryonic allantois fluid of chicken, which was then subjected to a 24 h inactivation at 37 °C and mixing with 1:1000 formaldehyde.

The chickens, which were randomized into 5 groups at 15 chicken per group, were subcutaneously vaccinated using one of the following: IBDV, Alum/IBDV (positive group), GRb1/IL-4-IBDV (IBDV:GRb1/IL-4 = 1:1, 0.2 mL), GRb1/IL-4@CS/CaP-IBDV (IBDV:GRb1/IL-4@CS/CaP = 1:1, 0.2 mL), CS/CaP-IBDV (IBDV:CS/CaP = 1:1, 0.2 mL) at the concentration of 1 mg/kg BW for GRb1, CS/CaP and GRb1/IL-4@CS/CaP, and IBDV, setting blank control group at the same time. On the 14 d, the chickens were subjected to subcutaneous injection at equal doses. On the 35 d, 42 d, with the initial immunization, harvesting of blood was accomplished. Treatment of all animals followed the provisions of the local Ethics Committee and the Chinese law.

### 2.5. Viability of DCs

DCs were prepared as previously reported [24]. Briefly, bone marrow-derived dendritic cells were isolated from femurs of 20-day-old SPF chickens, added to the cell culture plate to ensure that the final concentration of cells was 2 × 10^6^ mL, and cultured in a 37 °C incubator. They were cultured for two days, to gain the immature dendritic cells. After induction for 7 days, mature DCs were obtained. The prepared cell suspension was added into 96-well cell culture plates, 150 μL each. Then, GRb1/IL-4@CS/CaP-IBDV, CS/CaP-IBDV and GRb1 nanoparticles with different concentrations (0–500 μg/mL) were added, and cultured at 37 °C for 48 h. The MTT method was used to detect the cell activity of GRb1/IL-4@CS/CaP-IBDV, CS/CaP-IBDV and GRb1 against dendritic cells. The MTT detection method was used, as previously reported [25]. The OD value at 450 nm was measured by an enzyme-labeled instrument. The ratio of OD value in the experimental group to that in the control group was determined as cell viability. The relevant experiments were triplicated.

### 2.6. Investigation of DC Activation and Maturation, Cytokine Secretion in Bone Marrow Cells

Dendritic cells were collected and loaded into centrifuge tubes. After centrifugation, the supernatant was discarded. Cell precipitation was resuspended with 0.01 mol/L PBS and cell concentration was adjusted (2 × 10^6^). Anti-CD11c–Cy7, anti-MHC-II–APC as well as anti-CD80–FITC were used to stain the cells for 30 min in the dark at 4 °C, followed by thrice washing in PBS, fixation using 4% paraformaldehyde [26], and a final CytoFlex (Beckman CytoFlex) flow cytometry analysis. At the same time, ELISA was used to detect the expression levels of IL-1β and TNF-α in the cell supernatant. The relevant experiments were triplicated.

### 2.7. ELISA Analysis of Antibodies

On the 28 d, 42 d, enzyme-linked immunosorbent assay (ELISA) was employed for seral determination of the IBDV-specific IgG, IgG2a, and IgG1 among the vaccinated chickens. After coating the 96-well microplates using 1% IBDV lapping fluid in carbonate buffer, they were placed overnight under a 4 °C condition and then washed thrice using PBS containing Tween-20(PBST), blocked using 1% BSA in PBS, and subsequently subjected to a 2 h incubation under a 37 °C condition. This was followed by six times of washing and the addition of a sample at 100 μL per well for 1 h under a 37 °C condition. After another six times of washing, a further 1 h incubation proceeded at 37 °C using the HRP-labeled goat anti-chicken IgG, IgG2a, and IgG1 antibodies (diluted at 1:5000), followed again by six times of washing in PBS. Thereafter, each well was added with 100 μL of 3, 3′, 5, 5′-tetramethylbenzidine (TMB) solution to allow for a 10 min enzymatic reaction at ambient temperature, which was later terminated by the addition of stop solution (50 μL). A microplate reader was utilized to measure the OD450 nm (optical density at 450 nm), where all sample tests were replicated four times.

### 2.8. Determination of Cytokine Levels

The chicken serum was harvested separately 28 d and 42 d after immunization. We utilized the ready-to-use sandwich ELISA kits in order to assay the cytokine (IFN-γ, TNF-α, IL-4, IL-6, IL-1α, and IL-1β) expressions as per the protocol of the manufacturer.

### 2.9. Statistical Analysis

All the collected data are represented by GraphPad Prism 7.0 analysis software, and each group of data is represented by means ± SEM. Duncan’s multiple range test was employed for the significance analysis of statistical differences at a probability (*p*) level. *p* < 0.05 is the significant difference, represented by *, *p* < 0.01 for the difference is very significant, expressed as **, *p* < 0.001 for the difference is very significant, expressed as ***.

## 3. Results

### 3.1. Characterization of CS/CaP and GRb1/IL-4@CS/CaP Microparticles

**Figure 1:**
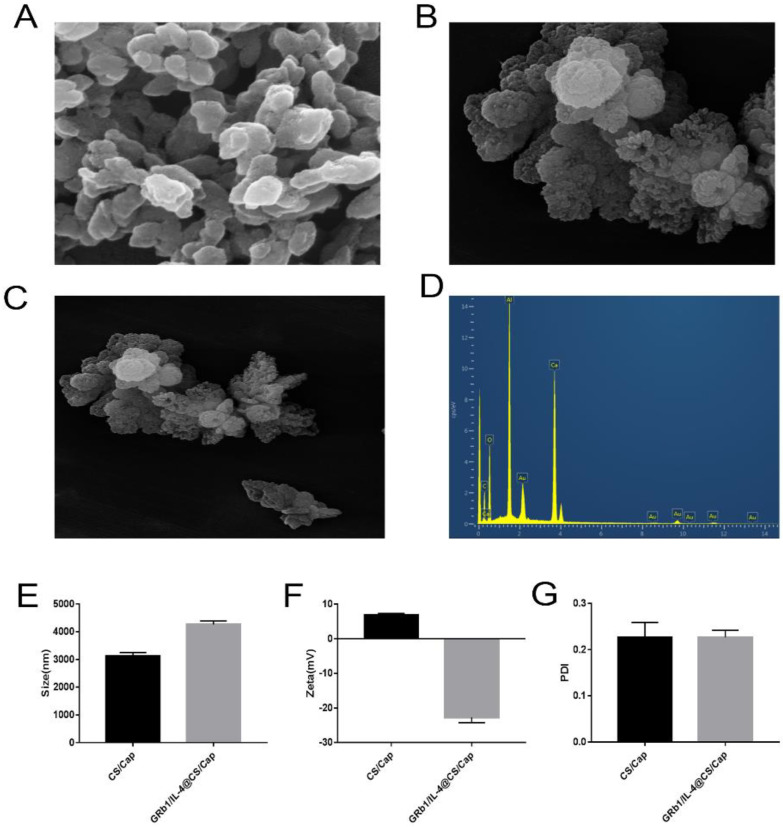
Characterization of CS/CaP and GRb1/IL-4@CS/CaP microparticles. (**A**–**C**) SEM of CS/CaP and GRb1/IL-4@CS/CaP. Scar bar = 5 μm. (**D**) Elemental mapping images. (**E**,**F**) Particle size and Zeta-potential of CS/CaP and GRb1/IL-4@CS/CaP. (**G**) PDI of CS/CaP and GRb1/IL-4@CS/CaP.

Scanning electron microscope (SEM) was adopted for investigating the morphology of CS/CaP and GRb1/IL-4@CS/CaP microparticles. Obviously, the grown microparticles reveal flower-like structures, as shown in Figure 1A–C. To further demonstrate the successfully prepared microparticles, the elements of GRb1/IL-4@CS/CaP microparticles were analyzed by Energy Dispersive Spectrometer (EDS), as shown in Figure 1D. the microemulsions contain Calcium (Ca), Carbon (C) and Oxygen( O) elements. The particle size and zeta potential of the samples can be found in Figure 1E,F. Besides, the mean hydrodynamic size of CS/CaP and GRb1/IL-4@CS/CaP was 3121 ± 72.46 nm and 4265 ± 72.92 nm, respectively. The average size of GRb1/IL-4@CS/CaP was slightly larger in comparison with unmodified CS/CaP, showing the successful modification of GRb1/IL-4 in the particle. The zeta potential for CS/CaP and GRb1/IL-4@CS/CaP was +6.87 ± 0.27 mV and −22.71 ± 0.89 mV, respectively. The values of the polydispersity index(PDI) were 0.265 ± 0.097, suggesting a narrow size distribution, as shown in Figure 1G.

### 3.2. Encapsulation Efficiency of CS/CaP and GRb1/IL-4@CS/CaP Microparticles

Microparticles were made by the assembly of nanoparticles, leading to a “flower” such as a structure with a rough surface that was conducive to the loading of GRb1 and IL-4. Through the encapsulation efficiency calculation, it was found that the loading efficiencies of GRb1 and IL-4 in GRb1/IL-4@CS/CaP were 49.52 ± 4.26% and 61.23 ± 2.31%, respectively.

### 3.3. Stability of CS/CaP and GRb1/IL-4@CS/CaP

**Figure 2:**
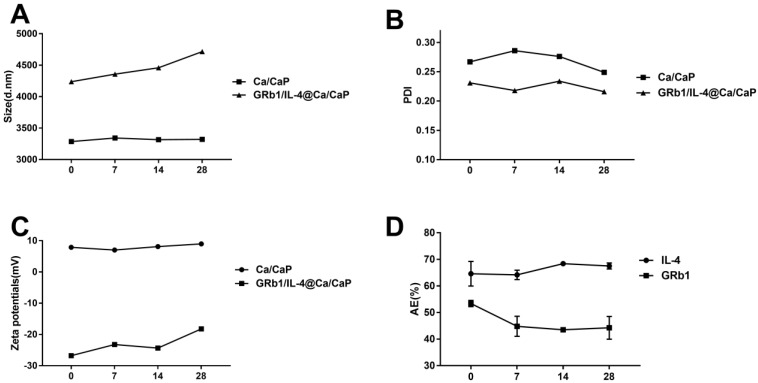
Stability of nanoparticles suspensions at 4 °C. The particle dimension (**A**), PDI value (**B**), zeta potential (**C**), as well as GRb1-AE (%) and IL-4-AE (%) (**D**) of the CCS/CaP and GRb1/IL-4@CS/CaP nanoparticles suspensions were assessed over a 4-week period.

For stability detection of the CS/CaP and GRb1/IL-4@CS/CaP nanoparticles suspensions, a 4-week period of measurement was accomplished for the particle dimension, zeta potential and PDI at 4 °C. According to Figure 2A–C, the mean CS/CaP dimensions varied between 3286 and 3321 nm over the 28 d, while the GRb1/IL-4@CS/CaP dimensions changed from 4237 nm to 4715 nm. The variations and deviations of the nanoparticle PDI values were indistinct, all of which were lower than 0.3 over the 4 weeks, indicating that the distributions of nanoparticles were all homogeneous in dimension. As shown in Figure 2D, the IL-4 (%) of GRb1/IL-4@CS/CaP ranged from 64.36% to 68.12%, and the GRb1 (%) of GRb1/IL-4@CS/CaP ranged from 52.37% to 41.28%. The IL-4 AE (%) of GRb1/IL-4@CS/Cap was stable over time and maintained at about 68%. The GRb1-AE (%) of GRb1/IL-4 @ CS/Cap decreased with time, lasting about 45%.

### 3.4. Viability of DCs

**Figure 3:**
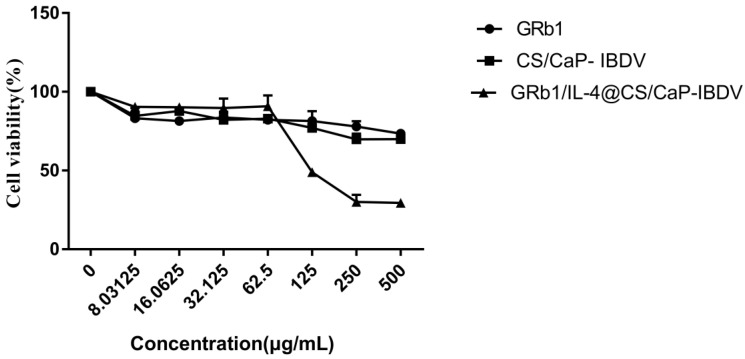
MTT assay was performed to determine the viability of dendritic cells (means ± SEMs, *n* = 6).

The cytotoxicity of the CS/CaP and GRb1/IL-4@CS/CaP against those cultivated with DCs was found with the application of the MTT assay. According to Figure 3, cell death was increased with an increase in concentration in GRb1/IL-4@CS/CaP (Figure 3). The result appeared to be 95.66% cell viability at the concentration of 62.5 μg/mL and lowered to 53.33% at the concentration of 125 μg/mL in GRb1/IL-4@CS/CaP groups. In addition, the cytotoxicity of CS/CaP on DCs was similar to the GRb1, The cytotoxicity of GRb1/IL-4@CS/CaP began to appear at the concentration of 125 µg/mL, and it had obvious cytotoxicity at the concentration of 500 µg/mL.

### 3.5. Surface Molecular Expression Levels and Cytokine Secretion in DCs

**Figure 4:**
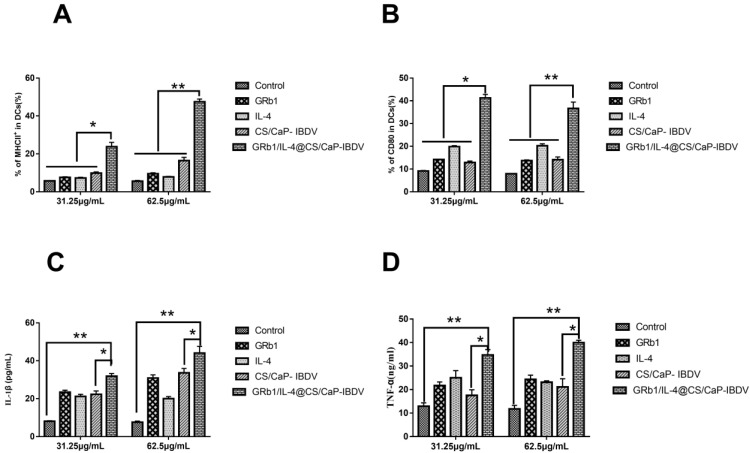
Effects of GRb1/IL-4@CS/CaP nanoparticles on the activation of DCs. The DCs levels of costimulatory molecules MHCII (**A**) and CD80 (**B**). The DCs-released IL-1β (**C**) and TNF-α (**D**) levels (* *p* < 0.05, ** *p* < 0.01).

The results demonstrated that DCs activation could upregulate the MHCII and CD80 of the surface molecules of DCs. The effect of GRb1/IL-4@CS/CaP on DCs was further determined. As shown in Figure 4A,B, compared with the control group, GRb1, IL-4 and CS/CaP groups, GRb1/IL-4@CS/CaP at the concentration of 31.25 μg/mL could significantly enhance the expression of MHCII and CD80 (*p* < 0.05), and 62.5 μg/mL GRb1/IL-4@CS/CaP could significantly enhance the expression of MHCII and CD80 (*p* < 0.01), and with a dose-dependent manner.

DCs were stimulated ex vivo with GRb1/IL-4@CS/CaP. At the same time, the levels of IL-1β and TNF-α were identified by applying ELISA assays in supernatant. In contrast to the CS/CaP, GRb1, IL-4 and control groups (*p* < 0.05 and *p* < 0.01), IL-1β and TNF-α releases were prompted in the GRb1/IL-4@CS/CaP group, showing dose responses (Figure 4C,D).

### 3.6. IBDV-Specific Antibody Response in Vaccinated Chicken

**Figure 5:**
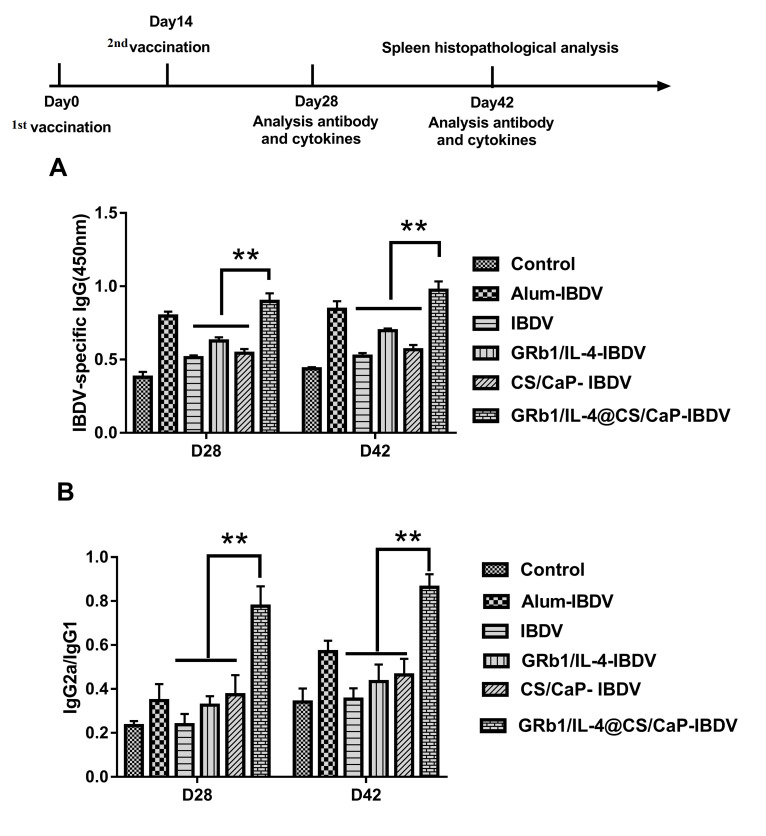
Chicken were subcutaneously vaccinated twice at a 2-week interval under varying vaccination programs. The chicken sera were harvested 28 d following the final vaccination after sacrifice. (**A**) IBDV-specific IgG output; (**B**) The IgG2a-to-IgG1 ratio. Data represented are means ± SEMs (** *p* < 0.01).

The adjuvant activity of GRb1 and IL-4 encapsulated in CS/CaP microparticles was explored by gathering serum from chicken immunized with IBDV as controls, GRb1/IL-4-IBDV, GRb1/IL-4@CS/CaP-IBDV, CS/CaP-IBDV. The Alum-IBDV was the positive group in the current experiment. According to Figure 5A, GRb1/IL-4@CS/CaP -IBDV exhibited a higher level of IBDV-specific IgG in comparison with the IBDV, GRb1/IL-4-IBDV and CS/CaP-IBDV groups. These results demonstrated that GRb1/IL-4@CS/CaP-IBDV could trigger more powerful IBDV-specific IgG immune responses.

By identifying the levels of IgG1 and IgG2a, it was proved that GRb1/IL-4@CS/CaP-IBDV has a regulatory effect on the immune response. Besides, the ratio of IgG2a/IgG1 was measured. Based on Figure 5B, treatment with GRb1/IL-4@CS/CaP -IBDV contributed to a higher ratio of IgG2a/IgG1 in comparison with others and higher than the IgG2a/IgG1 ratio of the Alum-IBDV group.

### 3.7. Cytokine Levels in Chicken Serum

**Figure 6:**
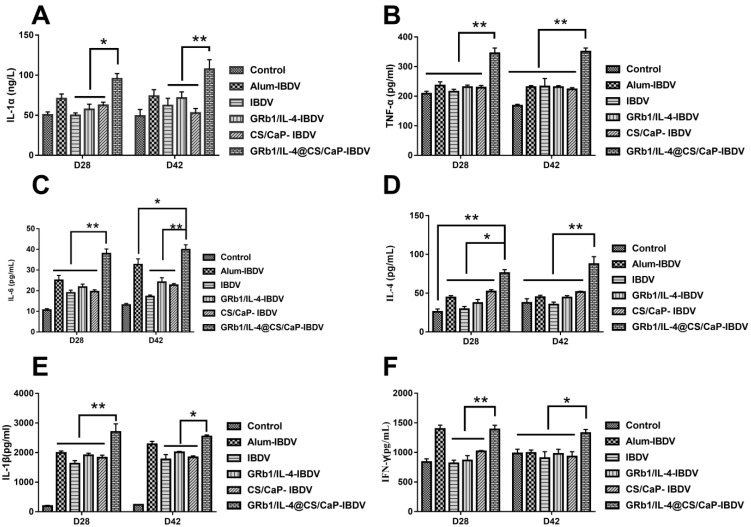
The chicken sera were harvested separately 28 d and 42 d following the final vaccination after sacrifice. The seral cytokine levels were determined. (**A**) IL-1α, (**B**) TNF-α, (**C**) IL-6, (**D**) IL-4, (**E**) IL-1β, (**F**) IFN-γ. Data represented are means ± SEMs (* *p* < 0.05, ** *p* < 0.01).

Cytokine release levels in serum are commonly used as a measure of macrophage activation [27]. Cytokine levels, IL-4(Th2), IL-6 (Th2), IFN-γ(Th1), and TNF-α (Th1), IL-1α, IL-1β in chicken serum were measured at 28 d and 42 d, aided by ELISA kits. Compared with the IBDV group, GRb1/IL-4-IBDV group and CS/CaP-IBDV group, at 28 d, the expression levels of IL-1α (Figure 6A), TNF-α (Figure 6B), IL-6 (Figure 6C) and IFN-γ (Figure 6F) in the GRb1/IL-4@CS/CaP-IBDV group were significantly increased (*p* < 0.01), as well as the extremely increased expression levels of IL-4 (Figure 6D) and IL-1β (Figure 6E) (*p* < 0.05). At 42 d, the expression levels of IL-1α (Figure 6A), TNF-α (Figure 6B), IL-6 (Figure 6C), and IL-4 (Figure 6D) in the GRb1/IL-4@CS/CaP-IBDV group were dramatically increased (*p* < 0.01), as well as the exceedingly increased expression levels of IL-1β (Figure 6E) and IFN-γ (Figure 6F) (*p* < 0.05), which were higher than those in the control group and the Alum-IBDV group.

## 4. Discussion

Adjuvants play an important role in enhancing and inducing humoral and cellular immunity. Therefore, the development of adjuvants, that can be safe and stable during the clinical vaccination process and enhance humoral and cellular immune responses, is an important part of the vaccine development process [28]. Calcium phosphate nanoparticles can transport antigens or drugs into the organism and specifically bind to the corresponding targeted sites, thereby improving the therapeutic effect. At the same time, the calcium phosphate matrix degrades relatively slowly, retaining the drug for a longer period of time after administration with fewer side effects. Taking calcium phosphate nanomaterials as an antigen or drug delivery system, we put ginsenoside Rb1/IL-4 in calcium phosphate nanoparticles to make the antigen carrier bind more effectively to the immune enhancer, prolonged the drug release rate, and provided a slow-release effect.

Ginsenosides play a good adjuvant role in animal immune responses and can significantly improve both the humoral and cellular immune responses [29]. Cellular IL-4 has been identified to process immunomodulatory effects on macrophage cells. Previous studies have revealed that CaP could foster a Th1-like immune response [30,31]. Similar to previous studies, the GRb1/IL-4@CS/CaP treatment could induce the activation of chicken dendritic cells (DCs), with the upregulated expression of MHCII and CD80, and the increased production of IL-1β and TNF-α. Dendritic cells play an important role in innate and acquired immunity. In the process of the immune response, dendritic cells play an important role in the interaction of surface MHC molecules with T and B lymphocytes. Cytokine secretion exerts important functions in initiating and regulating immune responses by immune cells, especially macrophages [32,33]. Taken together, GRb1/IL-4@CS/CaP could significantly improve the humoral and cellular immune response in chicken organisms.

In the past decades, traditional vaccine adjuvants have played an important role in the development of the avian vaccine. However, traditional vaccine adjuvants have several drawbacks, including adverse events, ineffective cellular immunity, as well as safety risks. A safe, effective and suitable adjuvant is important for current virus control efforts. In this study, the IBDV antigen was covalently coupled with GRb1/IL-4@CS/CaP microparticles to further investigate its potent function as an adjuvant. The results demonstrated that GRb1/IL-4@CS/CaP-IBDV could induce significantly higher levels of IBDV-specific IgG than other groups. Furthermore, it was proved that GRb1/IL-4@CS/CaP-IBDV had a regulatory effect on immune response, with a higher IgG2a/IgG1 ratio, and significantly increased the cytokines production in the serum, including IL-4, IL-6, IFN-γ, and TNF-α, IL-1α, IL-1β. When pathogens invade intracellular cells, Th1 cells exert a major effector cell function, while Th2 cells exert the main effector cell function under extracellular threat [34]. All findings indicated that GRb1/IL-4@CS/CaP nanoparticles could improve Th1 and Th2 immune responses, and process the ability to trigger stronger IBDV-specific IgG immune responses by promoting the enhancement of the Th1 pathway.

In summary, GRb1/IL-4@CS/CaP nanoparticles prepared by encapsulating GRb1/IL-4 into calcium phosphate nanoparticles can significantly improve humoral immune response and increase the expression of surface molecules of bone marrow-derived dendritic cells and secretion of cytokines. Therefore, GRb1/IL-4@CS/CaP was used as a delivery system in this study to provide a reference for further study on the mechanism of immune enhancement of GRb1 and its clinical application in animals.

## 5. Conclusions

In the present work, the parameters for efficiently fabricating GRb1/IL-4@CS/CaP particles were explored. The GRb1/IL-4@CS/CaP performed outstandingly in activating DCs. As suggested by our findings, GRb1/IL-4@CS/CaP may be useful as a scheme for efficiently adsorbing antigens and potently improving the DCs uptake of antigens. We probed deeper into the GRb1/IL-4@CS/CaP adjuvant action, and the results indicated that GRb1/IL-4@CS/CaP was capable of provoking a stronger antigen-specific IgG immune response when compared to the remaining groups. In particular, a hybrid Th1/Th2 response with Th1 bias could be triggered by the GRb1/IL-4@CS/CaP. Therefore, the GRb1/IL-4@CS/CaP delivery system can potentially trigger specific immune responses against infectious and viral diseases.

## Data Availability

Not applicable.

## Supplementary Materials

The following supporting information can be downloaded at: https://www.mdpi.com/article/10.3390/vetsci9070355/s1, Figure S1: Rb1 chemical structural formula.
