# Supplementary figures and images for: Chitosan/Calcium-Coated Ginsenoside Rb1 Phosphate Flower-like Microparticles as an Adjuvant to Enhance Immune Responses
Source: Vet Sci. 2022 Jul 13;9(7):355. doi: 10.3390/vetsci9070355 (PMC9316975; doi:10.3390/vetsci9070355)

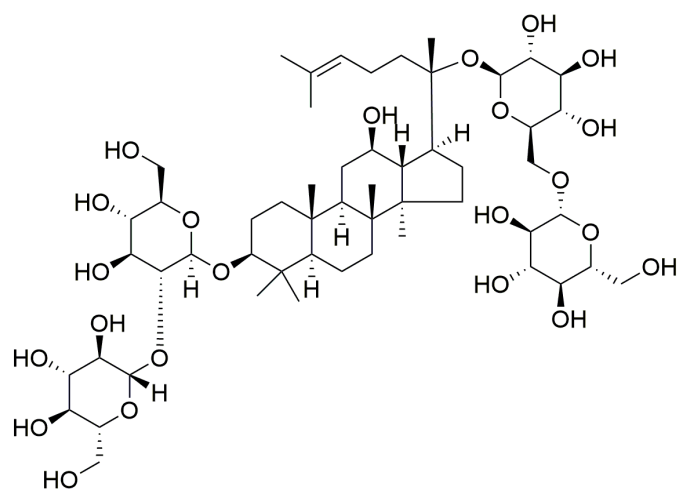

Supplement Figure S1. GRb1 chemical structural formula.

Supplement: Supplementary file 1 [file vetsci-09-00355-s001.zip › vetsci-1797466-supplementary.pdf]
